# Supplementary material for: Serum Cystatin-C is linked to increased prevalence of diabetes and higher risk of mortality in diverse middle-aged and older adults
Source: PLoS One. 2022 Sep 12;17(9):e0270289. doi: 10.1371/journal.pone.0270289 (PMC9467319; doi:10.1371/journal.pone.0270289)
Supplement: S1 File — (DOCX) [file pone.0270289.s001.docx]

**S1 Table: Mean and standard deviation of log cystatin-C values at visit 1 and visit 3 based on diabetes and death status.**

|  | **No diabetes Visit 1** | **Diabetes Visit 1** | **Dead Visit 1** | **Total** | **P-Value** |
| --- | --- | --- | --- | --- | --- |
| **Mean (SD)** |  |  |  |  |  |
| **Visit 1 Log Cystatin-C** | 0.00 (0.28) | 0.16 (0.43) | n/a | 0.03 (0.32) | P<0.001 |
| **Visit 3 Log Cystatin-C** | 0.08 (0.30) | 0.25 (0.41) | n/a | 0.11 (0.32) | P<0.001 |
|  | **No diabetes Visit 2** | **Diabetes Visit 2** | **Dead Visit 2** | **Total** | **P-Value** |
| **Mean (SD)** |  |  |  |  |  |
| **Visit 1 Log Cystatin-C** | -0.03 (0.25) | 0.06 (0.32) | 0.29 (0.47) | 0.03 (0.32) | P<0.001 |
| **Visit 3 Log Cystatin-C** | 0.07 (0.30) | 0.22 (0.38) | n/a | 0.11 (0.32) | P<0.001 |
|  | **No diabetes Visit 3** | **Diabetes Visit 3** | **Dead Visit 3** | **Total** | **P-Value** |
| **Mean (SD)** |  |  |  |  |  |
| **Visit 1 Log Cystatin-C** | -0.05 (0.23) | 0.01 (0.28) | 0.24 (0.44) | 0.03 (0.32) | P<0.001 |
| **Visit 3 Log Cystatin-C** | 0.07 (0.30) | 0.20 (0.36) | n/a | 0.11 (0.32) | P<0.001 |

**S2 Table: Counts and prevalence rates by diabetes and death status.**

|  | **Visit 2** | | | |  |
| --- | --- | --- | --- | --- | --- |
| **Visit 1** | **No diabetes** | **Diabetes** | **Dead** | **Total** | |
| **Unweighted N (%)** |  |  |  |  | |
| **No diabetes** | 4146 (98.70) | 427 (29.28) | 773 (66.46) | 5346 (78.90) | |
| **Diabetes** | 65 (1.30) | 1192 (70.72) | 424 (33.54) | 1681 (21.10) | |
|  | **Visit 3** | | | |  |
| **Visit 1** | **No diabetes** | **Diabetes** | **Dead** | **Total** | |
| **Unweighted N (%)** |  |  |  |  | |
| **No diabetes** | 3416 (98.87) | 568 (41.92) | 1362 (67.82) | 5346 (78.90) | |
| **Diabetes** | 52 (1.13) | 924 (58.08) | 705 (32.18) | 1681 (21.10) | |
|  | **Visit 3** | | | |  |
| **Visit 2** | **No diabetes** | **Diabetes** | **Dead** | **Total** | |
| **Unweighted N (%)** |  |  |  |  | |
| **No diabetes** | 3398 (98.39) | 282 (21.04) | 531 (25.95) | 4211 (63.62) | |
| **Diabetes** | 70 (1.61) | 1210 (78.96) | 339 (15.89) | 1619 (21.70) | |
| **Dead** | 0 (0.00) | 0 (0.00) | 1197 (58.16) | 1197 (14.68) | |

**S3 Table: Associations between eGFR and diabetes and death status at visit 1, visit 2, and visit 3.**

|  | **Visit 1 Diabetes** | | | |
| --- | --- | --- | --- | --- |
|  | **M0** | **M1** | **M2** | **M3** |
|  | **OR/CI** | **OR/CI** | **OR/CI** | **OR/CI** |
| **Normal** | ref | ref | ref | ref |
| **Mild** | 1.18 [0.98;1.41] | 1.14 [0.94;1.39] | 0.95 [0.78;1.16] | 0.97 [0.80;1.19] |
| **Moderate** | 2.60*** [2.17;3.11] | 2.43*** [1.98;3.00] | 1.69*** [1.33;2.13] | 1.68*** [1.32;2.15] |
|  | **Visit 2 Diabetes** | | | |
| **Normal** | ref | ref | ref | ref |
| **Mild** | 1.26* [1.05;1.51] | 1.22* [1.01;1.48] | 1.01 [0.82;1.25] | 1.03 [0.83;1.28] |
| **Moderate** | 2.11*** [1.77;2.52] | 2.01*** [1.65;2.44] | 1.31* [1.04;1.64] | 1.29* [1.00;1.65] |
|  | **Visit 2 Dead** | | | |
| **Normal** | ref | ref | ref | ref |
| **Mild** | 1.96*** [1.53;2.50] | 1.32 [0.99;1.75] | 1.29 [0.97;1.72] | 1.24 [0.93;1.66] |
| **Moderate** | 8.51*** [6.53;11.09] | 3.13*** [2.27;4.31] | 2.95*** [2.13;4.09] | 2.64*** [1.89;3.70] |
|  | **Visit 3 Diabetes** | | | |
| **Normal** | ref | ref | ref | ref |
| **Mild** | 1.22* [1.01;1.48] | 1.22 [0.98;1.51] | 1.02 [0.82;1.29] | 1.04 [0.83;1.31] |
| **Moderate** | 1.90*** [1.54;2.35] | 1.95*** [1.54;2.47] | 1.31 [1.00;1.73] | 1.32 [0.99;1.77] |
|  | **Visit 3 Dead** | | | |
| **Normal** | ref | ref | ref | ref |
| **Mild** | 2.12*** [1.77;2.56] | 1.48*** [1.19;1.84] | 1.39** [1.11;1.75] | 1.35* [1.07;1.70] |
| **Moderate** | 9.43*** [7.59;11.72] | 3.84*** [2.93;5.03] | 3.34*** [2.50;4.46] | 3.02*** [2.26;4.04] |

**Notes:**

eGFR= estimated Glomerular filtration rate; OR=Odds Ratio; CI=Confidence Interval; Normal= Normal kidney function; Mild = Mild kidney disfunction; Moderate = Moderate kidney disfunction

M0: No adjustment

M1: Age, sex, background, and education.

M2: M1 + BMI, smoking status, and drinking status.

M3: M2 + HDL, total cholesterol, and C-reactive protein.

**Note:** *: p<0.05. **: p<0.01. ***:p<0.001

**S4 Table: Test of modifications by race/ethnic background at visit 1, visit 2, and visit 3.**

| **Log Cystatin-C** | | | | | | | | | | | | |
| --- | --- | --- | --- | --- | --- | --- | --- | --- | --- | --- | --- | --- |
|  | **M0** | | | **M1** | | | **M2** | | | **M3** | | |
|  | **F** | **P-Value** | **df** | **F** | **P-Value** | **df** | **F** | **P-Value** | **df** | **F** | **P-Value** | **df** |
| **Diabetes Visit 1** | 0.979 | P=0.382 | 2.000 | 0.945 | P=0.395 | 2.000 | 0.186 | P=0.831 | 2.000 | 0.077 | P=0.926 | 2.000 |
| **Diabetes and Mortality Visit 2** | 4.927 | P=0.002 | 4.000 | 2.225 | P=0.079 | 4.000 | 1.691 | P=0.166 | 4.000 | 1.488 | P=0.219 | 4.000 |
| **Diabetes and Mortality Visit 3** | 3.147 | P=0.021 | 4.000 | 2.902 | P=0.030 | 4.000 | 2.125 | P=0.091 | 4.000 | 1.918 | P=0.121 | 4.000 |
| **eGFR** | | | | | | | | | | | | |
|  | **M0** | | | **M1** | | | **M2** | | | **M3** | | |
|  | **F** | **P-Value** | **df** | **F** | **P-Value** | **df** | **F** | **P-Value** | **df** | **F** | **P-Value** | **df** |
| **Diabetes Visit 1** | 1.554 | P=0.200 | 4.000 | 1.569 | P=0.196 | 4.000 | 1.048 | P=0.391 | 4.000 | 0.839 | P=0.507 | 4.000 |
| **Diabetes and Mortality Visit 2** | 1.476 | P=0.191 | 8.000 | 1.513 | P=0.177 | 8.000 | 1.508 | P=0.179 | 8.000 | 1.504 | P=0.180 | 8.000 |
| **Diabetes and Mortality Visit 3** | 1.551 | P=0.165 | 8.000 | 1.193 | P=0.323 | 8.000 | 1.021 | P=0.433 | 8.000 | 0.985 | P=0.459 | 8.000 |

**Notes:**

eGFR= estimated Glomerular filtration rate; OR=Odds Ratio; CI=Confidence Interval

M0: No adjustment

M1: Age, sex, background, and education.

M2: M1 + BMI, smoking status, and drinking status.

M3: M2 + HDL, total cholesterol, and C-reactive protein.

*: p<0.05. **: p<0.01. ***:p<0.001

**S5 Table:** **Interactions (main effects are omitted from Table) between log cystatin-C and race/ethnic background groups at visit 1, visit 2, and visit 3.**

|  | **Visit 1 Diabetes** | | | |
| --- | --- | --- | --- | --- |
|  | **M0** | **M1** | **M2** | **M3** |
|  | **OR/CI** | **OR/CI** | **OR/CI** | **OR/CI** |
| **White # Log Cystatin-C** | ref | ref | ref | ref |
| **Black # Log Cystatin-C** | 0.70 [0.41;1.17] | 0.73 [0.46;1.17] | 0.85 [0.51;1.43] | 0.91 [0.54;1.54] |
| **Latino # Log Cystatin-C** | 0.83 [0.42;1.64] | 0.82 [0.42;1.57] | 0.96 [0.52;1.78] | 1.03 [0.53;2.01] |
|  | **Visit 2 Diabetes** | | | |
| **White # Log Cystatin-C** | ref | ref | ref | ref |
| **Black # Log Cystatin-C** | 0.46* [0.24;0.87] | 0.47* [0.27;0.84] | 0.52* [0.28;0.95] | 0.53 [0.28;1.00] |
| **Latino # Log Cystatin-C** | 0.70 [0.32;1.52] | 0.63 [0.31;1.29] | 0.77 [0.35;1.70] | 0.89 [0.40;1.96] |
|  | **Visit 2 Dead** | | | |
| **White # Log Cystatin-C** | ref | ref | ref | ref |
| **Black # Log Cystatin-C** | 0.22*** [0.11;0.44] | 0.51* [0.27;0.96] | 0.52* [0.28;0.98] | 0.54 [0.29;1.02] |
| **Latino # Log Cystatin-C** | 0.81 [0.34;1.95] | 1.08 [0.47;2.49] | 1.13 [0.47;2.71] | 1.34 [0.54;3.27] |
|  | **Visit 3 Diabetes** | | | |
| **White # Log Cystatin-C** | ref | ref | ref | ref |
| **Black # Log Cystatin-C** | 0.51 [0.25;1.05] | 0.49* [0.25;0.93] | 0.49 [0.23;1.02] | 0.51 [0.24;1.08] |
| **Latino # Log Cystatin-C** | 0.37 [0.14;1.02] | 0.35* [0.14;0.90] | 0.43 [0.17;1.10] | 0.48 [0.19;1.21] |
|  | **Visit 3 Dead** | | | |
| **White # Log Cystatin-C** | ref | ref | ref | ref |
| **Black # Log Cystatin-C** | 0.33* [0.14;0.76] | 0.70 [0.30;1.62] | 0.76 [0.33;1.72] | 0.76 [0.34;1.72] |
| **Latino # Log Cystatin-C** | 0.89 [0.29;2.70] | 0.96 [0.37;2.54] | 1.06 [0.39;2.86] | 1.22 [0.44;3.41] |

**Notes:**

eGFR= estimated Glomerular filtration rate; OR=Odds Ratio; CI=Confidence Interval; Log=natural logarithm

M0: No adjustment

M1: Age, sex, background, and education.

M2: M1 + BMI, smoking status, and drinking status.

M3: M2 + HDL, total cholesterol, and C-reactive protein.

**Note:** *: p<0.05. **: p<0.01. ***:p<0.001

**S6 Table: Visit 1 descriptive characteristics by sex.**

|  | **Male** | **Female** | **Total** | **P-Value** |
| --- | --- | --- | --- | --- |
| **Unweighted N** | 2,946 | 4,081 | 7027 |  |
| **Weighted %** | 45.7 | 54.3 |  |  |
| **Background** |  |  |  |  |
| White | 86.31 | 83.80 | 84.97 | P<0.001 |
| Black | 7.63 | 9.88 | 8.83 |  |
| Latino | 6.06 | 6.32 | 6.20 |  |
| **Education** |  |  |  |  |
| <12 years | 17.04 | 17.14 | 17.09 | P<0.001 |
| 12 years | 29.74 | 36.61 | 33.40 |  |
| >12 years | 53.22 | 46.25 | 49.50 |  |
| **Drinker** |  |  |  |  |
| Not Drinker | 37.19 | 50.94 | 44.53 | P<0.001 |
| Drinker | 62.81 | 49.06 | 55.47 |  |
| **Smoker** |  |  |  |  |
| Not Smoker | 32.04 | 51.45 | 42.39 | P<0.001 |
| Smoker | 67.96 | 48.55 | 57.61 |  |
| **Diabetes V1** |  |  |  |  |
| No Diabetes | 78.06 | 79.64 | 78.90 | P=0.137 |
| Diabetes | 21.94 | 20.36 | 21.10 |  |
| **Diabetes and dead V2** | |  |  |  |
| No Diabetes | 60.97 | 65.94 | 63.62 | P<0.001 |
| Diabetes | 23.89 | 19.78 | 21.70 |  |
| Dead | 15.13 | 14.28 | 14.68 |  |
| **Diabetes and dead V3** | |  |  |  |
| No diabetes | 50.67 | 55.88 | 53.45 | P=0.003 |
| Diabetes | 22.69 | 20.11 | 21.31 |  |
| Dead | 26.64 | 24.01 | 25.24 |  |
| **HDL** |  |  |  |  |
| Low | 73.43 | 71.00 | 72.14 | P=0.064 |
| Normal | 26.57 | 29.00 | 27.86 |  |
| **eGFR Visit 1†** |  |  |  |  |
| Normal | 31.88 | 33.42 | 32.70 | P=0.024 |
| Mild | 46.02 | 42.08 | 43.92 |  |
| Moderate | 22.11 | 24.50 | 23.38 |  |
| **eGFR Visit 2†** |  |  |  |  |
| Normal | 25.08 | 25.31 | 25.21 | P=0.341 |
| Mild | 44.51 | 42.50 | 43.42 |  |
| Moderate | 30.41 | 32.19 | 31.37 |  |
| **eGFR Visit 3†** |  |  |  |  |
| Normal | 23.31 | 21.90 | 22.54 | P=0.158 |
| Mild | 44.49 | 43.31 | 43.85 |  |
| Moderate | 32.20 | 34.79 | 33.60 |  |
| **Mean (SD)** |  |  |  |  |
| **Age** | 65.60 (5.01) | 66.65 (10.45) | 66.16 (7.78) | P<0.001 |
| **BMI** | 28.61 (5.01) | 28.53 (6.71) | 28.57 (5.93) | P=0.615 |
| **Cholesterol (mg/dL)** | 196.09 (37.34) | 207.77 (42.18) | 202.32 (40.32) | P<0.001 |
| **CRP (mg/L)** | 2.08 (3.88) | 2.58 (4.60) | 2.35 (4.27) | P=0.001 |
| **Log Cystatin-C V1** | 0.03 (0.28) | 0.04 (0.36) | 0.03 (0.32) | P=0.060 |
| **Log Cystatin-C V2** | 0.08 (0.31) | 0.09 (0.36) | 0.08 (0.33) | P=0.332 |
| **Log Cystatin-C V3** | 0.10 (0.30) | 0.11 (0.34) | 0.11 (0.32) | P=0.264 |

**Notes:**

BMI=Body Mass Index; HDL=High Density Lipoprotein; CRP=C-reactive protein. eGFR= estimated Glomerular filtration rate; Log= natural logarithm

**†:** Normal= Normal kidney function; Mild = Mild kidney disfunction; Moderate = Moderate kidney disfunction

**S7 Table: Test for modifications by sex at visit 1, visit 2, and visit 3.**

| **Log Cystatin-C** | | | | | | | | | | | | | |
| --- | --- | --- | --- | --- | --- | --- | --- | --- | --- | --- | --- | --- | --- |
|  | **M0** | | | **M1** | | | **M2** | | | **M3** | | |  |
|  | **F** | **P-Value** | **df** | **F** | **P-Value** | **df** | **F** | **P-Value** | **df** | **F** | **P-Value** | **df** |  |
| **Diabetes Visit 1** | 0.046 | P=0.831 | 1.000 | 0.088 | P=0.767 | 1.000 | 0.000 | P=0.988 | 1.000 | 0.017 | P=0.895 | 1.000 |  |
| **Diabetes and Mortality Visit 2** | 1.946 | P=0.153 | 2.000 | 0.844 | P=0.435 | 2.000 | 0.490 | P=0.616 | 2.000 | 0.427 | P=0.654 | 2.000 |  |
| **Diabetes and Mortality Visit 3** | 3.349 | P=0.042 | 2.000 | 2.480 | P=0.093 | 2.000 | 2.083 | P=0.134 | 2.000 | 1.943 | P=0.153 | 2.000 |  |
| **eGFR** | | | | | | | | | | | | | |
|  | **M0** | | | **M1** | | | **M2** | | | **M3** | | |  |
|  | **F** | **P-Value** | **df** | **F** | **P-Value** | **df** | **F** | **P-Value** | **df** | **F** | **P-Value** | **df** |  |
| **Diabetes Visit 1** | 1.120 | P=0.334 | 2.000 | 0.984 | P=0.380 | 2.000 | 1.120 | P=0.334 | 2.000 | 1.053 | P=0.356 | 2.000 |  |
| **Diabetes and Mortality Visit 2** | 0.644 | P=0.633 | 4.000 | 1.024 | P=0.404 | 4.000 | 0.644 | P=0.633 | 4.000 | 1.643 | P=0.177 | 4.000 |  |
| **Diabetes and Mortality Visit 3** | 0.539 | P=0.708 | 4.000 | 1.009 | P=0.411 | 4.000 | 0.539 | P=0.708 | 4.000 | 1.588 | P=0.191 | 4.000 |  |

**Notes:**

eGFR= estimated Glomerular filtration rate; OR=Odds Ratio; CI=Confidence Interval

M0: No adjustment

M1: Age, sex, background, and education.

M2: M1 + BMI, smoking status, and drinking status.

M3: M2 + HDL, total cholesterol, and C-reactive protein.

**S8 Table:** **Associations between eGFR (including <15 in the moderate group) and diabetes and death status at visits 1, visit 2, and visit 3.**

|  | **Visit 1 Diabetes** | | | |
| --- | --- | --- | --- | --- |
|  | **M0** | **M1** | **M2** | **M3** |
|  | **OR/CI** | **OR/CI** | **OR/CI** | **OR/CI** |
| **Normal** | 1.00 [1.00;1.00] | 1.00 [1.00;1.00] | 1.00 [1.00;1.00] | 1.00 [1.00;1.00] |
| **Mild** | 1.18 [0.98;1.41] | 1.14 [0.94;1.39] | 0.96 [0.79;1.17] | 0.98 [0.80;1.19] |
| **Moderate** | 2.69*** [2.25;3.20] | 2.52*** [2.06;3.09] | 1.76*** [1.40;2.20] | 1.75*** [1.38;2.21] |
|  | **Visit 2 Diabetes** | | | |
| **Normal** | 1.00 [1.00;1.00] | 1.00 [1.00;1.00] | 1.00 [1.00;1.00] | 1.00 [1.00;1.00] |
| **Mild** | 1.26* [1.05;1.51] | 1.22* [1.01;1.48] | 1.01 [0.82;1.25] | 1.03 [0.83;1.28] |
| **Moderate** | 2.13*** [1.78;2.55] | 2.02*** [1.66;2.46] | 1.32* [1.05;1.65] | 1.29* [1.00;1.66] |
|  | **Visit 2 Dead** | | | |
| **Normal** | 1.00 [1.00;1.00] | 1.00 [1.00;1.00] | 1.00 [1.00;1.00] | 1.00 [1.00;1.00] |
| **Mild** | 1.96*** [1.53;2.50] | 1.33* [1.00;1.76] | 1.30 [0.98;1.72] | 1.25 [0.94;1.67] |
| **Moderate** | 8.97*** [6.93;11.61] | 3.37*** [2.48;4.58] | 3.18*** [2.33;4.34] | 2.82*** [2.05;3.90] |
|  | **Visit 3 Diabetes** | | | |
| **Normal** | 1.00 [1.00;1.00] | 1.00 [1.00;1.00] | 1.00 [1.00;1.00] | 1.00 [1.00;1.00] |
| **Mild** | 1.22* [1.01;1.48] | 1.22 [0.98;1.51] | 1.03 [0.82;1.29] | 1.04 [0.83;1.31] |
| **Moderate** | 1.90*** [1.54;2.35] | 1.94*** [1.53;2.45] | 1.31 [1.00;1.72] | 1.32 [0.99;1.75] |
|  | **Visit 3 Dead** | | | |
| **Normal** | 1.00 [1.00;1.00] | 1.00 [1.00;1.00] | 1.00 [1.00;1.00] | 1.00 [1.00;1.00] |
| **Mild** | 2.12*** [1.77;2.56] | 1.49*** [1.20;1.85] | 1.40** [1.11;1.76] | 1.36* [1.07;1.71] |
| **Moderate** | 9.85*** [7.97;12.18] | 4.09*** [3.15;5.30] | 3.56*** [2.70;4.70] | 3.19*** [2.41;4.23] |

**Notes:**

eGFR= estimated Glomerular filtration rate; OR=Odds Ratio; CI=Confidence Interval; Log=natural logarithm; Normal= Normal kidney function; Mild = Mild kidney disfunction; Moderate = Moderate kidney disfunction

M0: No adjustment

M1: Age, sex, background, and education.

M2: M1 + BMI, smoking status, and drinking status.

M3: M2 + HDL, total cholesterol, and C-reactive protein.

**S1 Fig: Visits flowchart.**


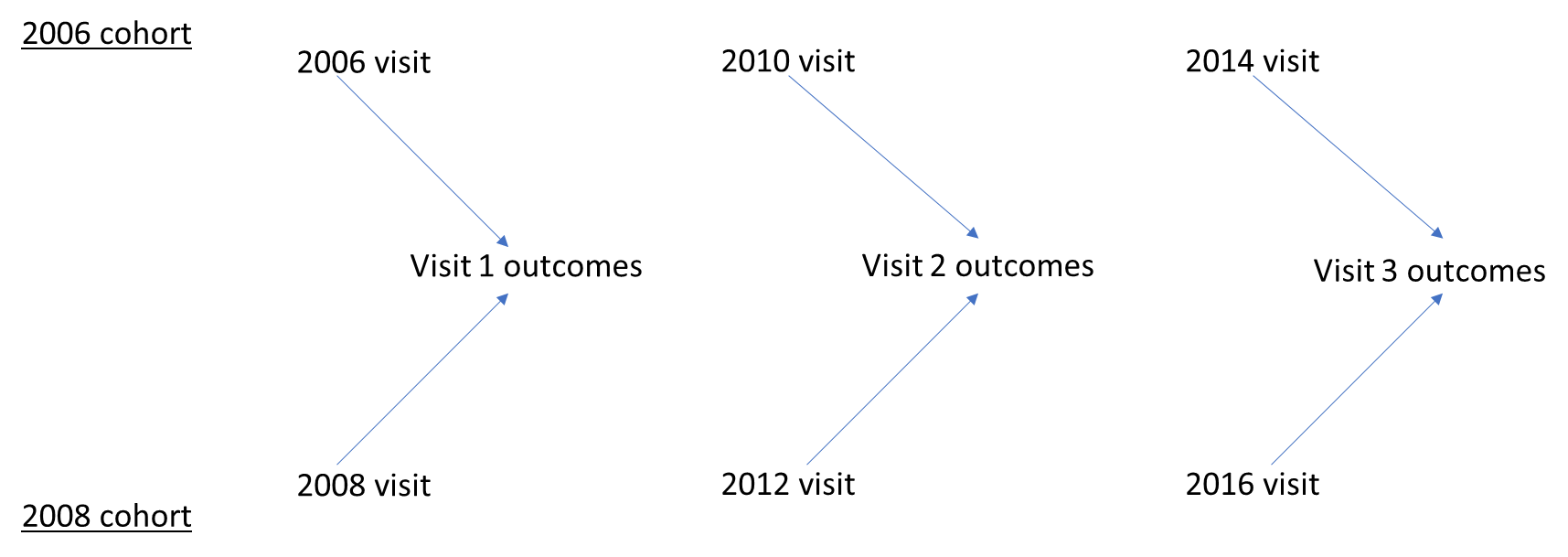


**S2 Fig: Analytic sample flowchart.**

Participated in 2006 or 2008 biomarker study

N=13,064

50+ years

N=12,725

Excluded

N=339

Identified as Latino, Black, or White

N=12,461

Excluded

N=264

Participated in biomarker visit 2 or reported deceased

N=9,858

Excluded

N=2,603

No

No

No

Analytical Sample

N= 7,027

Participated in biomarker visit 2 or reported deceased

N=8,117

Excluded

N=1,741

No

Participated in biomarker visit 2 or reported deceased

N=7,027

Excluded

N=1,090

No

**S3 Fig: Prevalence (marginal probability and 95% confidence interval) of no diabetes, diabetes, and death status at visit 1, visit 2, and visit 3 by Cystatin-C stratified by background.**


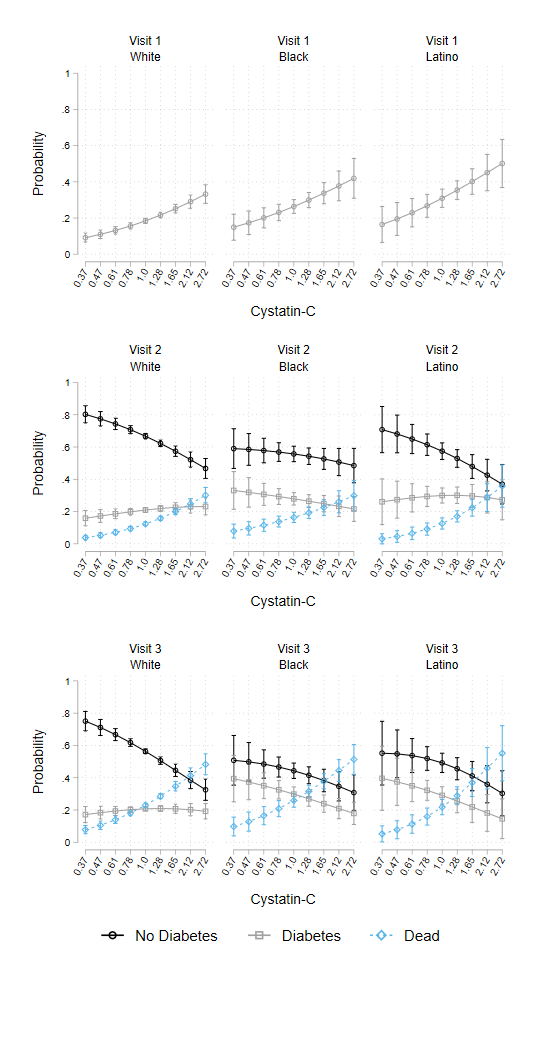


**Notes:** Models include for age, sex, education, background, high density lipoprotein, total cholesterol, body mass index, smoking status, C-reactive protein, and drinking status.

**S4 Fig:** **Prevalence (marginal probability and 95% confidence interval) of no diabetes, diabetes, and death status at visit 1, visit 2, and visit 3 by eGFR stratified by background.**


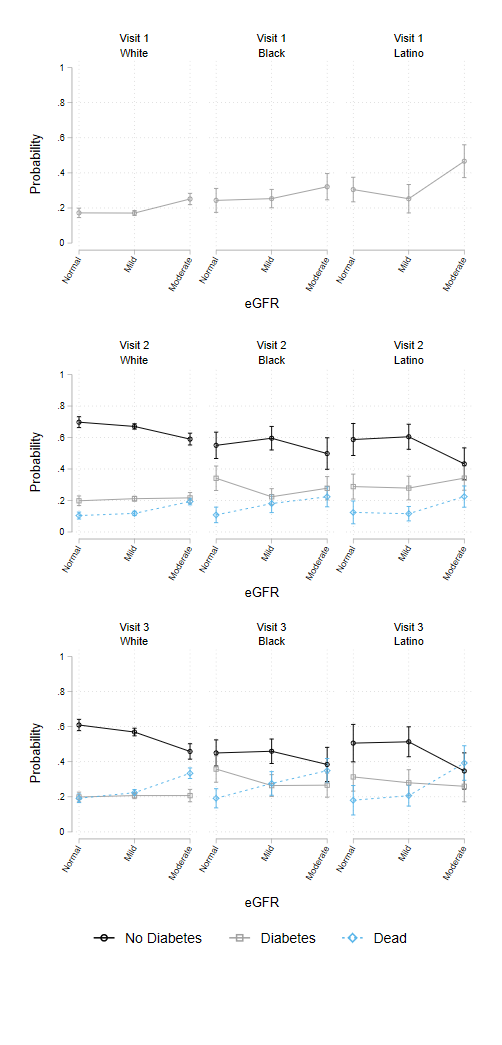


**Notes:** GFR=Glomerular filtration rate; Normal= Normal kidney function; Mild = Mild kidney disfunction; Moderate = Moderate kidney disfunction

Models include for age, sex, education, background, high density lipoprotein, total cholesterol, body mass index, smoking status, C-reactive protein, and drinking status.

**S5 Fig:** **Prevalence (marginal probability and 95% confidence interval) of no diabetes, diabetes, death status at Visits 1, 2, and 3 by Cystatin-C stratified by sex.**


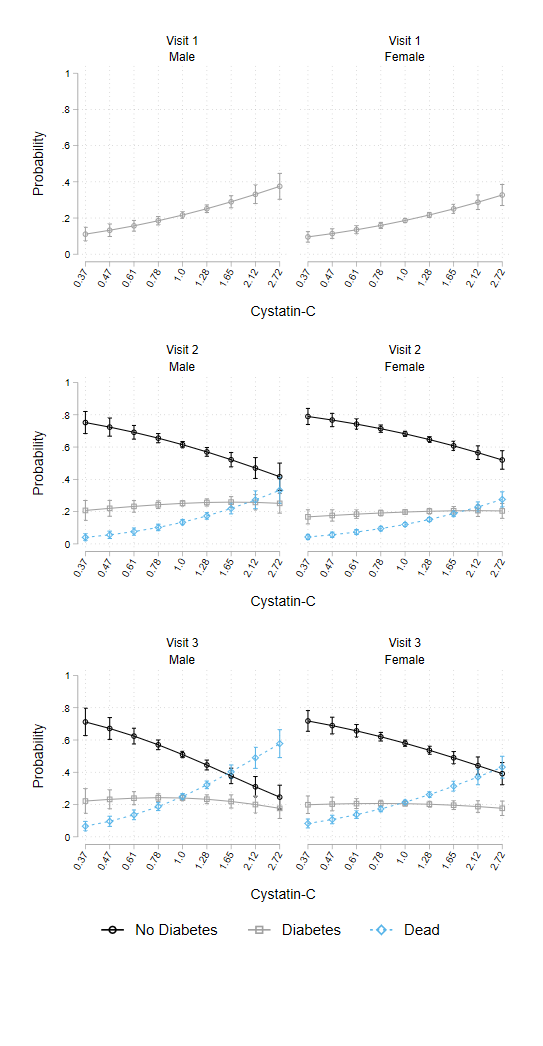


**Notes:** Models include for age, sex, education, background, high density lipoprotein, total cholesterol, body mass index, smoking status, C-reactive protein, and drinking status.

**S6 Fig: Prevalence (marginal probability and 95% confidence interval) of no diabetes, diabetes, and death status at Visits 1, 2, and 3 by estimated GFR stratified by sex.**


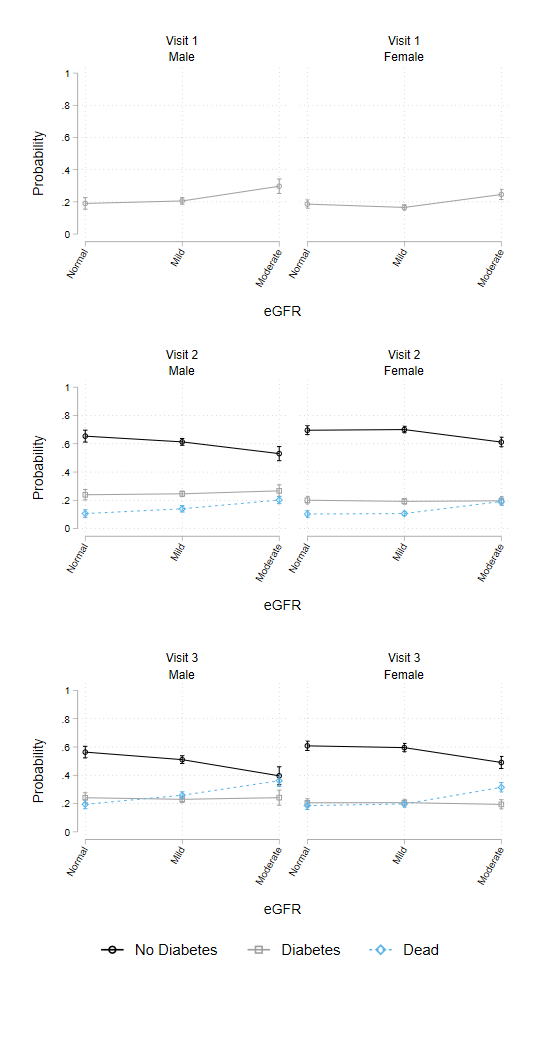


**Notes:** GFR=Glomerular filtration rate; Normal= Normal kidney function; Mild = Mild kidney disfunction; Moderate = Moderate kidney disfunction

Models include for age, sex, education, background, high density lipoprotein, total cholesterol, body mass index, smoking status, C-reactive protein, and drinking status.

**S1 Data: HRS biomarker and RAND dataset addresses.**

**Datasets used**

**1) Rand Files**
2006 HRS RAND data available at <https://hrsdata.isr.umich.edu/data-products/2006-rand-hrs-fat-file>

2008 HRS RAND data available at <https://hrsdata.isr.umich.edu/data-products/2008-rand-hrs-fat-file>

2010 HRS RAND data available at <https://hrsdata.isr.umich.edu/data-products/2010-rand-hrs-fat-file>

2012 HRS RAND data available at <https://hrsdata.isr.umich.edu/data-products/2012-rand-hrs-fat-file>

2014 HRS RAND data available at <https://hrsdata.isr.umich.edu/data-products/2014-rand-hrs-fat-file>

2016 HRS RAND data available at <https://hrsdata.isr.umich.edu/data-products/2016-rand-hrs-fat-file>


**2) Biomarker files**
2006 HRS Biomarker data available at <https://hrsdata.isr.umich.edu/data-products/2006-biomarker-data>

2008 HRS Biomarker data available at <https://hrsdata.isr.umich.edu/data-products/2008-biomarker-data>

2010 HRS Biomarker data available at <https://hrsdata.isr.umich.edu/data-products/2010-biomarker-data>

2012 HRS Biomarker data available at <https://hrsdata.isr.umich.edu/data-products/2012-biomarker-data>

2014 HRS Biomarker data available at <https://hrsdata.isr.umich.edu/data-products/2014-biomarker-data>

2016 HRS Biomarker data available at <https://hrsdata.isr.umich.edu/data-products/2016-biomarker-data>
