# Supplementary material for: Serum Cystatin-C is linked to increased prevalence of diabetes and higher risk of mortality in diverse middle-aged and older adults
Source: PLoS One. 2022 Sep 12;17(9):e0270289. doi: 10.1371/journal.pone.0270289 (PMC9467319; doi:10.1371/journal.pone.0270289)
Supplement: Attachment — Submitted filename: Reviews.docx [file pone.0270289.s002.docx]

5. Review Comments to the Author

Please use the space provided to explain your answers to the questions above. You may also include additional comments for the author, including concerns about dual publication, research ethics, or publication ethics. (Please upload your review as an attachment if it exceeds 20,000 characters)

Reviewer #1: Excellent job in analyzing this large data, this would be considered a well done manuscript for future references. I do have few points as below:

1. Line 51: No mention of diabetes as a risk factor of cardiovascular disease and death, while knowing that cardiovascular disease is the most common cause of death in patients with diabetes, hence relevant to noticed increased relative risk of death among patients with elevated Cystatin-C.

We thank the reviewer for pointing us to this omission and have now addressed this in the introduction (lines 90-96).

2. Line 59: Hba1c better to written (henceforth) as HbA1c or HbA1c.

We have updated the manuscript to consistently use HbA1c throughout the manuscript.

3. Authors could spend more time discussing and possibly explaining the reason of elevated Cystatin-C and increased risk of diabetes and death in terms of pathophysiology.

Thank you for your comment. We have included language to discuss pathophysiology (lines 298-307).

Reviewer #2: Whilst the study has an advantage of a relatively large sample size and longitudinal design, it lacks originality and novelty. An extensive literature on the associations of Cystatin C with mortality and DM risk including various ethnic groups already exists (examples of some of those studies not referenced in the manuscript:

Shlipak MG, Wassel Fyr CL, Chertow GM, Harris TB, Kritchevsky SB, Tylavsky FA, Satterfield S, Cummings SR, Newman AB, Fried LF. Cystatin C and mortality risk in the elderly: the health, aging, and body composition study. J Am Soc Nephrol. 2006 Jan;

Peralta CA, Lee A, Odden MC, Lopez L, Zeki Al Hazzouri A, Neuhaus J, Haan MN. Association between chronic kidney disease detected using creatinine and cystatin C and death and cardiovascular events in elderly Mexican Americans: the Sacramento Area Latino Study on Aging. J Am Geriatr Soc. 2013

Sabanayagam C et all. Serum cystatin C and prediabetes in non-obese US adults. Eur J Epidemiol. 2013 Apr;

K. Sahakyan et all. Serum Cystatin C and the Incidence of Type 2 Diabetes Mellitus, Diabetologia 2011)

Thank you, we now reference these manuscripts in the introduction section (90-105) and distinguish our manuscript.

The manuscript does not read well in sections, particularly the results section, which needs to be presented in a clearer manner. Also, I would suggest placing the tables with the description of the findings within Results Section rather than Statistical Analyses. There are a number of small errors/typos throughout this this manuscript which needs correcting such as:

Thank you we have revised for better clarity. In addition, we have conducted a thorough copy edit to fix aberrant errors and typos.
Line 132- error in eGRF categories – fixed (now lines 150-51)
HDL categories- discrepancy in description on text/table 1 - fixed
Table 1- units of measure in the table
Line 240 - unclear what authors mean by “lower association”. Updated text in ms
Duplication of reference: 11=13 - fixed


Please justify the choice of covariates on statistical grounds (unless biologically plausible mechanism linking covariate to the dependant variable exists) and provide the cut-off points for the categorical variables where missing.

Thank you. We now include a rationale for choice of covariates. Additionally, we included cutoff for categorical variables.

The authors might want to take advantage of the availability of the Cystatin C data at three time points and apply more complex statistics such as repeated measures GLM to allow more robust longitudinal analysis.

Unfortunately, repeated measures GLM, or longitudinal generalized mixed models would not be appropriate in our case given that death is included as a competing risk (category) in our outcomes. Mixed modeling for complex survey sampling like the HRS is also further complicated by the limited capabilities to account for the sampling design including stratification, clustering and appropriate weighting (particularly weighting the different levels). Accounting for death independently, would require more sophisticated techniques, such as joint growth and discrete-time survival which go well beyond the scope of this analyses. We believe that our methodology is valid and we restrict our inferences accordingly in text and discussion. We now add this rationale to the limitations section and point to the need for use of longitudinal models as future considerations. (Lines 343-354)
